# A high biodiversity mitigates the impact of ocean acidification on hard-bottom ecosystems

**DOI:** 10.1038/s41598-020-59886-4

**Published:** 2020-02-19

**Authors:** Eugenio Rastelli, Bruna Petani, Cinzia Corinaldesi, Antonio Dell’Anno, Marco Lo Martire, Carlo Cerrano, Roberto Danovaro

**Affiliations:** 1 Stazione Zoologica Anton Dohrn, Villa Comunale, 80121 Naples, Italy; 2 Department of Ecology, Agronomy and Aquaculture, University of Zadar, 23000 Zadar, Croatia; 3 Department of Materials, Environmental Sciences and Urban Planning, Polytechnic University of Marche, 60131 Ancona, Italy; 4 Department of Life and Environmental Sciences, Polytechnic University of Marche, 60131 Ancona, Italy

**Keywords:** Biodiversity, Biogeochemistry, Marine biology

## Abstract

Biodiversity loss and climate change simultaneously threaten marine ecosystems, yet their interactions remain largely unknown. Ocean acidification severely affects a wide variety of marine organisms and recent studies have predicted major impacts at the pH conditions expected for 2100. However, despite the renowned interdependence between biodiversity and ecosystem functioning, the hypothesis that the species’ response to ocean acidification could differ based on the biodiversity of the natural multispecies assemblages in which they live remains untested. Here, using experimentally controlled conditions, we investigated the impact of acidification on key habitat-forming organisms (including corals, sponges and macroalgae) and associated microbes in hard-bottom assemblages characterised by different biodiversity levels. Our results indicate that, at higher biodiversity, the impact of acidification on otherwise highly vulnerable key organisms can be reduced by 50 to >90%, depending on the species. Here we show that such a positive effect of a higher biodiversity can be associated with higher availability of food resources and healthy microbe-host associations, overall increasing host resistance to acidification, while contrasting harmful outbreaks of opportunistic microbes. Given the climate change scenarios predicted for the future, we conclude that biodiversity conservation of hard-bottom ecosystems is fundamental also for mitigating the impacts of ocean acidification.

## Introduction

Ocean acidification (OA) caused by the ongoing increase in anthropogenic carbon dioxide (CO_2_) emissions represents one of the most important threats to marine biodiversity and ecosystem functioning^1–5^. Seawater acidification alters the carbonates system and affects the metabolism and physiology of calcifying marine species, leading to lower calcification rates and to malformation or dissolution of carbonate structures (e.g., organisms’ skeletons)^2–11^. These can in turn determine changes in the biotic potential, mobility, survival rates and can increase the risk of their extinction^2–11^. Changes in species abundance, composition and functions can alter ecosystem’s properties^12–17^, with impacts comparable or higher than other drivers of environmental change^17–19^. Moreover, whilst all species contribute to ecosystem functions, changes in the abundance of keystone species, ecosystem engineers and habitat forming species can have much larger cascading effects than the loss of other species^20,21^. For instance, the local extinction of habitat forming species have been shown to drive significant detrimental effects, in terms of habitat complexity, functional redundancy and ecosystem resilience^22–25^. Ocean acidification can thus alter ecosystem stability to a different extent, depending on the impact on specific taxa; the higher the functional role of the impacted taxa, the higher the possibility that OA will reduce or compromise key ecosystem functions^26–29^.

Increasing evidence indicates that the ecological interactions among the different components of multispecies assemblages can influence the response of each single species to environmental change^30–36^. This implies that the effects of OA observed in experiments conducted on a single species (i.e., one species isolated from its assemblages and habitat) might be different than those we can observe when the species is within complex networks of multispecific interactions, such as those occurring in their natural environment^30,31^.

So far, experimental studies tested only single species response to OA, while the impacts on multispecies assemblages remains untested. Moreover, whilst it is known that microbes play a key role in the functioning of marine ecosystems^37–39^ and on their host species^40–42^, only scarce information is available on the impact of OA on the microbe-host interactions in marine organisms.

In order to provide new insights on the impact of OA on marine life, we used as a model for our experiments, the coralligenous assemblages, which are formed by calcareous biogenic structures produced by calcifying algae and invertebrates, which are an hot spot of benthic biodiversity (including a large number of habitat forming species, such as the red coral *C. rubrum* and several calcifying algae and sponges) in several temperate seas^43–46^. These habitats are extremely vulnerable to fluctuations in environmental conditions, and the presence of calcareous structures makes these systems particularly threatened by warming and OA^43–54^. In the present study, we conducted long-term time-course acidification experiments (using the scenario of 1000 ppm of atmospheric CO_2_ predicted by 2100^4^), to test for the responses of the red coral and of the entire coralligenous assemblage to OA. We tested the effects in terms of growth rates, and in terms of microbe-host associations. The analyses conducted on a Mediterranean coralligenous habitat indicate that increasing biodiversity values enable a stronger resistance to the impact of OA.

## Methods

### Experimental set-up

*Corallium rubrum* apical branches (n = 90, about 4 cm long with ca 200 polyps each, sampled from different coral colonies) and natural assemblages (n = 36 blocks of coralligenous assemblages, standardized to a volume of ca. 1 dm^3^ each) were collected at 30 ± 0.5 m depth in the Marine Protected Area of Portofino (Ligurian Sea, Italy). The sampling effort was selected based on previous experience to assure an optimal level of experimental replication, at the same time avoiding excess harvesting to minimize any potential impacts of sampling on the local population of the target species^2^. The experimental set-up followed the procedures described in ref. ^2^ with some modifications, as follows. After collection, all corals and natural coralligenous assemblages were placed in 50-L tanks containing air-bubbled seawater, filtered and maintained at the *in situ* temperature of 13.6 ± 0.5 °C and salinity of 37.5 ± 0.5. Constant temperature conditions were assured by continuous monitoring using a YSI TDS conductivity meter during the short transfer to the laboratory (6 hours). After 10 months of acclimation at the *in situ* temperature and salinity, the coral colonies and coralligenous assemblages were transferred to mesocosms filled with 10 L of 20-µm filtered seawater (collected *in situ*). To test the impact of OA according to the IPCC scenario of OA for the end of this century^4^, control mesocosms were exposed to current pCO_2_ levels (i.e., ca 400 ppm CO_2_) and an equal number of mesocosms were exposed to 1000 ppm CO_2_, reaching a final pH of approx. 7.7. To test the role of biodiversity in the response to OA, we included a first series of 6 mesocosms (3 controls and 3 acidified) containing *C. rubrum* alone, and a second series of 12 mesocosms (6 controls and 6 acidified) containing *C. rubrum* in association with coralligenous assemblages of 6 different values of biodiversity (i.e., replicated assemblages composed of organisms representing 6, 7, 8, 9, 10 or 11 different families). The biodiversity of the different systems analyzed was assessed considering the dominant organisms (i.e., the taxa covering more than 1% of the total area of the coralligenous blocks). In each mesocosm, five colonies of *C. rubrum* were grouped, either alone (in the first series of mesocosms) or in association with three blocks of coralligenous assemblages for each of the 6 different biodiversity treatments tested (in the second series). Despite 10 days have been already reported to be sufficient to detect negative impacts of acidification on *C. rubrum*^2^, we extended here the duration of the experiment to 86 days to increase the possibility to appreciate consistent effects on the red coral as well as on other potentially vulnerable coralligenous species.

A computerized Bronkhorst High-Tech BV series mass flow meter was used to acidify seawater by air-CO_2_ gas mixture bubbling at 1000 ppm CO_2_^2^, while controls received bubbling at 400 ppm CO_2_. The flow rate was increased slowly up to final 150 ml min^−1^ to gradually reach the desired pH values in all mesocosms. All coral colonies and coralligenous assemblages survived during the whole experiment and after. Mesocosms were placed in a large tank with water supplied by three pumps, which re-circulated the water through the chiller (TECO SeaChill TR5) and within the water bath assuring homogeneous physical-chemical properties of the water mass inside each tank to avoid small-scale temperature or pH gradients. Seawater within each mesocosm was re-circulated continuously at the rate of 190 L h^−1^, with renewal of 20-µm filtered seawater (collected *in situ*) ensured for the entire duration of the experiment. Mesocosms were shaded to reproduce *in situ* light conditions from the selected depth using an opaque cloth, and partially covered with plastic wrap to facilitate equilibration between the gas mixtures and the experimental seawater and to minimize water evaporation. All mesocosms were fed three times a week with 2 mL of 10^4^ *Artemia salina* nauplii and 2 mL of 10^5^ microalgae (*Nannochloropsis* sp. and *Tetraselmis* sp.) in 20-µm filtered seawater.

### Physical-chemical variables and carbonate parameters

During the experimental run, temperature and salinity were measured daily with an YSI TDS conductivity meter. pH was determined daily using a Crison pH electrode/meter calibrated with NBS buffers (accuracy ± 0.005). Alkalinity was determined at 8 different time points (at the start of the experiment and every approximately 1–2 weeks), by using an open-cell potentiometric titration procedure calibrated with certificated alkalinity standards^55^. Seawater samples (500 ml) for Total Alkalinity (TA) were preserved with 200 μl of 50% HgCl_2_ saturated solution to avoid any biological alteration and were stored in the dark at 4 °C until analysis. TA was determined on ~140 g subsamples using a titration system comprising of a 250 ml open titration cell thermo-regulated at 25 ± 0.1 °C, a Crison pH electrode/meter calibrated with certified DIN 19267 pH/mV standards (±0.5 mV) and a Crison Burette 1S (±0.001ml). The accuracy of the titrations was ±3 μmol kg^−1^. Parameters of the carbonate system, including pCO_2_, CO_3_^2−^, HCO_3_^−^ and DIC concentrations, saturation of aragonite (Ω_Ar_) and calcite (Ω_Ca_) were estimated from the measured values of pH, TA, temperature, salinity, phosphate and silicate concentrations using the program CO2Sys.xls 2011^56^. The concentration of proteins, carbohydrates, and lipids was measured spectrophotometrically^57^. The sum of the carbohydrate, protein and lipid concentrations converted into carbon equivalents (by using the conversion factors of 0.40, 0.49 and 0.75 μg C μg^−1^, respectively) was defined as biopolymeric carbon (BPC)^57,58^.

### Analysis of the coralligenous assemblages

The biodiversity and the areal coverage of the benthic organisms of the coralligenous assemblages was inspected using a standardized photographic protocol and photoQuad software system for advanced image processing^59^. The photographic analysis was confirmed through parallel stereomicroscope identification of each component of the coralligenous assemblages^60,61^. The shift in the areal coverage of sponges and macroalgae in control and acidified mesocosms was calculated as the difference in the relative coverage (as % of the total surface area of the coralligenous) of each taxon, between the end and the start of the experiment. The net growth rates of the *C. rubrum* colonies, as well as the coral sclerites’ accretion, skeletal morphology and polyps’ activity, were determined using standard methods as previously described^2^. Briefly, the net growth rates of the *C. rubrum* colonies was measured using a buoyant weighting method^62^, a highly accurate technique for the determination of mass increase in living corals^63^. All coral colonies were suspended at aquaria filled with 20 µm filtered seawater (T = 13.6 °C, Sal = ~37.7) by a hook attached to an aluminum wire hanging from a Radwag bottom-loading scale to minimize the effect on the physiology and health of the coral colonies. The dry weights of the axial skeleton, of the sclerites and of the total skeleton (i.e., axial skeleton plus sclerites) were also determined. *C. rubrum* growth rates were calculated as the percentage weight difference (measured as buoyant weight) between the beginning and the end of the experiment, and normalized to the coral branch initial weight^64^. To calculate the mean basal diameter growth rate, first buoyant weight were converted into dry weights using the linear regression equation between buoyant weights and total (i.e., axial skeleton plus sclerites) dry skeleton weights^65^. Subsequently, dry weights were converted to basal diameters using the following relationship: W = 0.086 × G^2.198^. Where W = dry weight; G = basal diameter of the colony^64^. The annual basal diameter growth rates were finally calculated by normalizing to the duration of the interval (n = 86 days) and multiplying by 365. The accuracy and reliability of the adopted protocols for determining the growth rates was previously tested^2^, and confirmed also in the present experiments. The buoyant weight of the red corals in treated and control systems was checked to be significantly related with their axial skeleton dry weight and their total skeleton dry weight (i.e., scleraxis plus sclerites) (Supplementary Fig. S1).

To detect the effects of acidification on the sclerites accretion of *C. rubrum*, a calcein-labeling experiment was conducted^2^. At the end of the experiment, one coral colony from each replicated mesocosm was transferred in separated aquaria and kept under same conditions of temperature and pH as from where the colonies were withdrawn. An aliquot of calcein (Sigma C-0875, with a final concentration of 10 mg L^−1^) was added into each separated aquarium (treated and untreated coral branches) 120 h before the end of the bubbling experiment. Afterwards, their apical parts were soaked in 12% solution of sodium hypochlorite for 24 h, until all organic material was removed^66^. *C. rubrum* calcifies more rapidly in its apical regions where the sclerites are directly incorporated to form the medullar part of the axial skeleton^67^. The sclerites were then rinsed several times with reagent grade water (MilliQ), mounted on slides and analyzed under epifluorescence microscopy (excitation filter 450–490 nm) for total and fluorescent capstan and cross sclerites counting. The relative abundance of fluorescent (cross and capstan) sclerites was used as an estimate of newly-accreted calcium carbonate skeletal elements^2^. In addition, values of the fluorescent cross to capstan sclerites ratio were used to evaluate differences in the production of the two different skeleton elements among treatments.

To detect the effects of acidification on calcification, coral colonies were prepared for observation under Scanning Electron Microscope (SEM), analyzing the fine-scale skeletal morphology. At the end of the experiment, one colony was withdrawn from each mesocosm and the apical branches of the colonies were treated to remove all organic material using the same method adopted for sclerites analyses (described above). Further, skeletal samples were mounted on aluminum stubs using carbon adhesive tabs and subsequently coated with gold/palladium (Au/Pb) for five minutes using a Polaron Range sputter coater. SEM observations were conducted with a Philips® XL 20 microscope to assess the presence of skeletal abnormalities at low pH (e.g., malformation or dissolution of the skeleton). In addition, to quantify the possible effect of pH on the sclerites we used SEM in combination with a 0–2 visual rank-scale approach for determining the percentage of dissolution-damage^68^. Rank 0 corresponded to 0% damage, rank 1 corresponded to 0 < damage < 50%, and rank 2 corresponded to >50% damage, analyzing at least 100 sclerites per colony^68^.

The activity of each colony was assessed by determining the state of activity of its polyps (and expressed as the prevailing state of the polyps’ expansion)^2^. Each coral colony was assigned to three different expansion states of the polyp’s body as follows: prevailing number of totally expanded polyp and tentacles (value 2), tentacles or polyps emerging from the gastric cavity (value 1) and totally retracted polyps (value 0). The polyps’ activity was expressed as the percentage of polyps at the maximum expansion state relative to the total number of polyps. In addition, the percentage of open polyps per each colony was determined^2^. Only polyps of similar size were considered and the smallest polyps were excluded to avoid overestimating the number of retracted polyps. The air-CO_2_ gas mixtures were pumped carefully far from the coral colonies into the aquaria to avoid confounding effects on polyps’ activity.

### Microbiological analyses

To check for the lack of pathogenic outbreaks potentially occurring in our mesocosms, virus and prokaryote counts from seawater samples of each mesocosm during the experiment were conducted by epifluorescence microscopy and SYBR Green I staining^69^.

Catalysed Reporter Deposition Fluorescence *In Situ* Hybridization (CARD-FISH) was conducted for the quantification of calcibacteria in the sponge *H. columella*. Briefly, calcibacteria were extracted from 1 g of fresh sponge tissue as previously described^70^. Aliquots of the calcibacteria extracts obtained and of the fresh sponge tissue^71^ were analysed by CARD-FISH^72^ using Horseradish Peroxidase (HRP)-labeled probes Eub-mix (Eub338, Eub338-II and Eub338-III) targeting Bacteria. The absence of nonspecific signals was routinely checked using the NON-338 probe^72^. Hybridization was performed at 35 °C for 2 hours, followed by washing of the hybridized samples into preheated (37 °C) washing buffer and treatment for Cy3-tyramide signal amplification (at 37 °C in the dark) and probe stabilization in PBS buffer (pH 7.6) amended with Triton X-100 (final concentration, 0.05%). Filters were then observed under epifluorescence microscopy under green light.

The presence of fungi was checked by scraping areas of the natural coralligenous substrates, followed by direct detection of fungi by calcofluor staining^73^. The frequency of fungal detection was calculated as the percentage of scraped spots (of standard surface area) in which fungi were detected, relative to the total number of spots analyzed.

### Statistical analyses

To test for differences in the investigated variables between different experimental treatments, analysis of variance was carried out. In detail, a two-way analysis of variance was used to test for significant differences in the growth rates of the different coralligenous taxa between treatments (i.e., acidified vs control pH conditions) and to test for any potential tank effects (i.e., including “mesocosm” as a random factor, nested within the factor “treatment”)^3^.

The differences in the feeding behaviour, sclerite calcification and cross-to-capstan ratio were assessed using a two-way analysis of variance including factors treatment (acidified vs control conditions) and incubation type (red coral alone or in association with multispecies assemblages).

A distance-based permutational multivariate analysis of variance^74^ was used to test for significant differences in the integrity of the red coral sclerites (capstans and crosses), including factors treatment (acidified vs control), time (initial vs final time) and incubation type (red coral alone or with multispecies assemblages).

To assess the effect of acidification on the association between the sponge *Hemimycale columella* and calcibacteria, a three-way analysis of variance was used including factors treatment (acidified vs control), time (initial vs final) and biodiversity level (2 levels: low, i.e, multispecies assemblages including organisms of 6 families; and high, i.e., multispecies assemblages including organisms of 11 families).

A three-way analysis of variance was used to assess potential differences in the prokaryotic and viral abundance in the mesocosms during the experiment, including factors treatment (acidified vs control), time (initial, mid-term and final time) and incubation type (red coral alone or with multispecies assemblages).

To exclude potential differences in temperature and salinity among mesocosms during the experiment and to assess the significant changes in pH, total alkalinity and seawater carbonate system due to the acidification treatment, a mixed-design analysis of variance was used, including factors treatment (acidified vs control), time (86 subsequent days) and incubation type (red coral alone or with multispecies assemblages).

All analyses of variance were conducted on Euclidean distance similarity matrices, after checking the homogeneity of variance using the Cochran’s test. Post-hoc tests were carried out when significant differences were encountered. All statistical analyses were carried out using R^75^ and Primer 6+ software^76^.

## Results and Discussion

All of the acidified systems reached the predicted seawater pH values, decreasing by approximately 0.4 units compared to the controls (Table 1 and Supplementary Fig. S2). This mimics with high accuracy the conditions assumed to occur in the future by the IPCC scenario of 1000 ppm of atmospheric CO_2_^4^ and it is consistent with previous acidification experiments on the same population of *C. rubrum*^2^. The analysis of the available historical data^77^ highlights a significant trend of acidification of the water masses in the marine area from which the organisms were sampled (Supplementary Fig. S3). Indeed, we found in this site evidence of a decrease of −0.0025 pH units per year (Supplementary Fig. S3), which is consistent with current observations of the generalized trend of acidification for the whole Mediterranean basin^78,79^. This suggests that, in the future, this area might face longer periods at the low pH conditions tested in our study (i.e., pH < 7.8), highlighting the urgent need to understand the possible impacts of such environmental changes on these coralligenous assemblages and on the processes able to influence their response to OA.

**Table 1:** Physical-chemical variables and main carbonate parameters measured *in situ* and in control or acidified treatments.

System		S	T (°C)	pH	TA	DIC	pCO_2_	HCO_3_^−^	CO_3_^2−^	ΩAr	ΩCa
					(µmol kg^−1^)	(µmol kg^−1^)	(µatm)	(µmol kg^−1^)	(µmol kg^−1^)		
*in situ conditions*		13.6 ± 0.5	38.1 ± 0.2	8.06 ± 0.01	2586 ± 4	2332 ± 3	416 ± 1	2115 ± 3	210 ± 4	2.72 ± 0.01	4.17 ± 0.01
Control (86 days average)	Coral alone	13.5 ± 0.1	37.6 ± 0.1	8.14 ± 0.01	2699 ± 61	2399 ± 53	362 ± 9	2169 ± 46	217 ± 8	4.80 ± 0.19	3.31 ± 0.13
	Coral + Coralligenous	13.6 ± 0.1	37.5 ± 0.1	8.13 ± 0.01	2648 ± 60	2357 ± 53	363 ± 9	2135 ± 47	208 ± 7	4.62 ± 0.16	3.18 ± 0.11
Acidified Treatment (86 days average)	Coral alone	13.4 ± 0.1	37.5 ± 0.1	7.84 ± 0.01	2843 ± 35	2669 ± 13	818 ± 97	2494 ± 21	144 ± 21	3.19 ± 0.46	2.20 ± 0.31
	Coral + Coralligenous	13.6 ± 0.1	37.6 ± 0.1	7.84 ± 0.01	2790 ± 37	2621 ± 20	807 ± 87	2451 ± 24	139 ± 19	3.09 ± 0.42	2.13 ± 0.29
Acidified Treatment (on day 86)	Coral alone	13.4 ± 0.2	37.8 ± 0.2	7.74 ± 0.01	2767 ± 50	2663 ± 39	1064 ± 33	2522 ± 33	100 ± 7	2.21 ± 0.16	1.53 ± 0.11
	Coral + Coralligenous	13.4 ± 0.2	37.8 ± 0.1	7.74 ± 0.01	2692 ± 87	2588 ± 82	1020 ± 24	2451 ± 76	98 ± 5	2.18 ± 0.11	1.50 ± 0.08

For the controls and acidified treatments, reported are salinity (S); temperature (T); total alkalinity (TA); dissolved inorganic carbon (DIC); partial pressure of carbon dioxide (pCO_2_); bicarbonates concentration (HCO_3_^−^); carbonates concentration (CO_3_^2−^); aragonite saturation state (Ω_Ar_); calcite saturation state (Ω_Ca_).

In our acidified treatments, the carbonate parameters reflected the experimentally induced increase in pCO_2,_ which reached final 1042 ± 36 µatm versus control values of 362 ± 9 µatm. The acidification treatment determined a significant decrease of average Ω_Ar_ compared with the controls (2.2 ± 0.1 *versus* 4.7 ± 0.2), Ω_Ca_ (1.5 ± 0.1 *vs*. 3.2 ± 0.1) and CO_3_^2−^ (99 ± 5 *vs*. 212 ± 8 µmol kg^−1^), and an increase in DIC (2626 ± 70 *vs*. 2378 ± 53 µmol kg^−1^) and HCO_3_^−^ (2487 ± 66 *vs*. 2152 ± 47 µmol kg^−1^) compared to the controls (p < 0.01; Table 1 and Supplementary Fig. S2). All mesocosms displayed oversaturation of aragonite and calcite in the seawater and consistent values for the rest of the seawater physical-chemical variables during the entire duration of the experiments (Table 1 and Supplementary Fig. S2). These results confirm current evidences that, despite the high acidification levels, the Mediterranean waters are still largely oversaturated in both calcite and aragonite^79^.

Besides the red coral *C. rubrum* (Family *Coralliidae*), the dominant organisms of the coralligenous multispecies assemblages utilised in the present study belonged to the following families: *Hildenbrandiaceae* and *Hapalidiaceae* (algae), *Hymedesmiidae*, *Ancorinidae*, *Clathrinidae*, *Leucosoleniidae* and *Sycettidae* (sponges), *Celleporidae*, *Smittinidae*, *Beanidae*, *Crisiidae* and *Schizoporellidae* (bryozoans), *Dendrophylliidae and Epizoanthidae* (cnidarians), and *Serpuloidae* (polychaetes). The most abundant taxa in the coralligenous multispecies assemblages we tested were sponges and algae. Sponges were the dominant metazoa, accounting on average for 71 ± 23% of the surface covered by macrozoobenthos. Two main classes of Porifera were present: *Calcarea* (calcifying sponges including the species *Sycon ciliatum*, *Leucosolenia sp*., and *Clathrina sp*.), characterized by a calcareous skeleton, and *Demospongiae*, mainly characterized by a siliceous skeleton. Demosponges included two main groups: epilithic sponges (*Haplosclerida n.c*., *Suberitidae*, *Aplysina sp*., *Phorbas sp*., *Hymedesmia sp*., *Dictyonella sp*., *Haliclona* (Gellius) *marismedi*, *Hemimycale columella* Bowerbank, 1874 and endolithic sponges (*Dercitus* (Stoeba) *plicatus* and *Jaspis incrustans*). *H. columella* represented >94% of the total sponge coverage. The macroalgae included two main groups: turf algae (non-calcifying and mainly represented by *Hildenbrandia cf. rubra* -Sommerfelt- Meneghini, 1841) and crustose coralline algae (calcifying algae, almost entirely represented by *Phymatolithon sp*. Foslie, 1898).

As the red coral *C. rubrum*, also the dominant algae (*Phymatolithon sp.)* and sponge (*H. columella)* in the coralligenous assemblages we tested have been previously shown to be affected by low-pH seawater values^2,3,7,80,81^ and thus represented optimal models for our multispecies acidification experiment.

**Figure 1:**
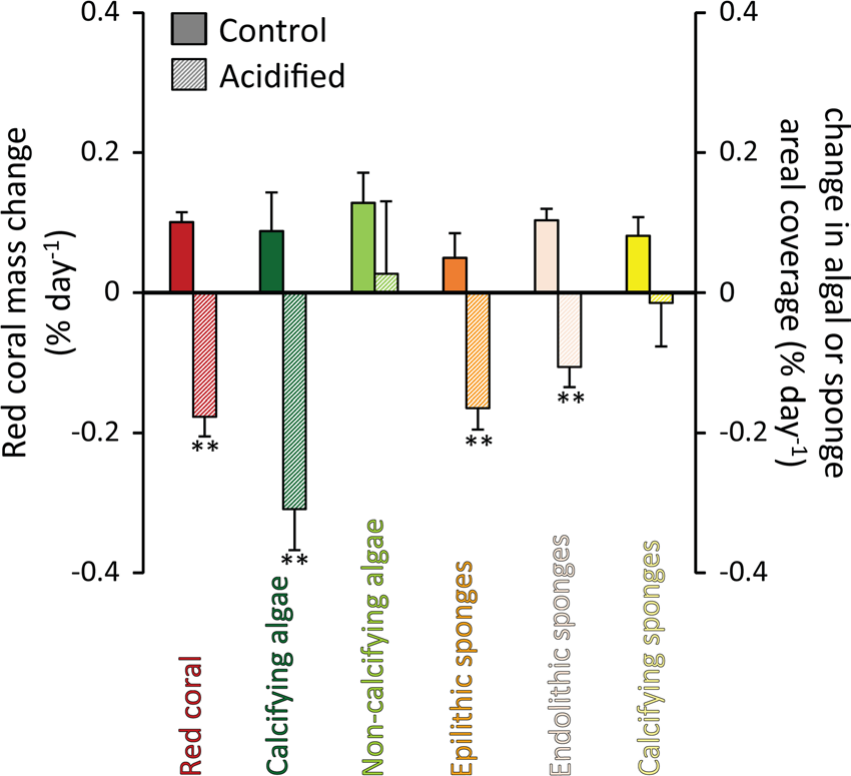
Impact of acidification on the dominant taxa. Reported are the changes in the growth rates of different coralligenous taxa due to the acidification treatment. The growth rates are expressed as shifts in mass weight for the red coral or in areal coverage for macroalgae and sponges. Reported are average values and SDs. Asterisks indicate significant differences (**p < 0.01) in the acidified treatment compared with the respective control.

The acidification treatment significantly reduced the growth rates of different taxa compared to the controls (Fig. 1), determining a negative shift in the *C. rubrum* mass weight (by −0.18 ± 0.03% d^−1^, compared with control values of 0.10 ± 0.01% d^−1^) and a decrease in the areal coverage of other dominant organisms of the coralligenous assemblages (including calcifying algae, endolithic sponges and epilithic sponges; respectively by −0.31 ± 0.06, −0.16 ± 0.09 and −0.11 ± 0.03% d^−1^, compared with control values of 0.09 ± 0.06, 0.05 ± 0.03 and 0.10 ± 0.02% d^−1^; p < 0.01; Fig. 1).

**Figure 2:**
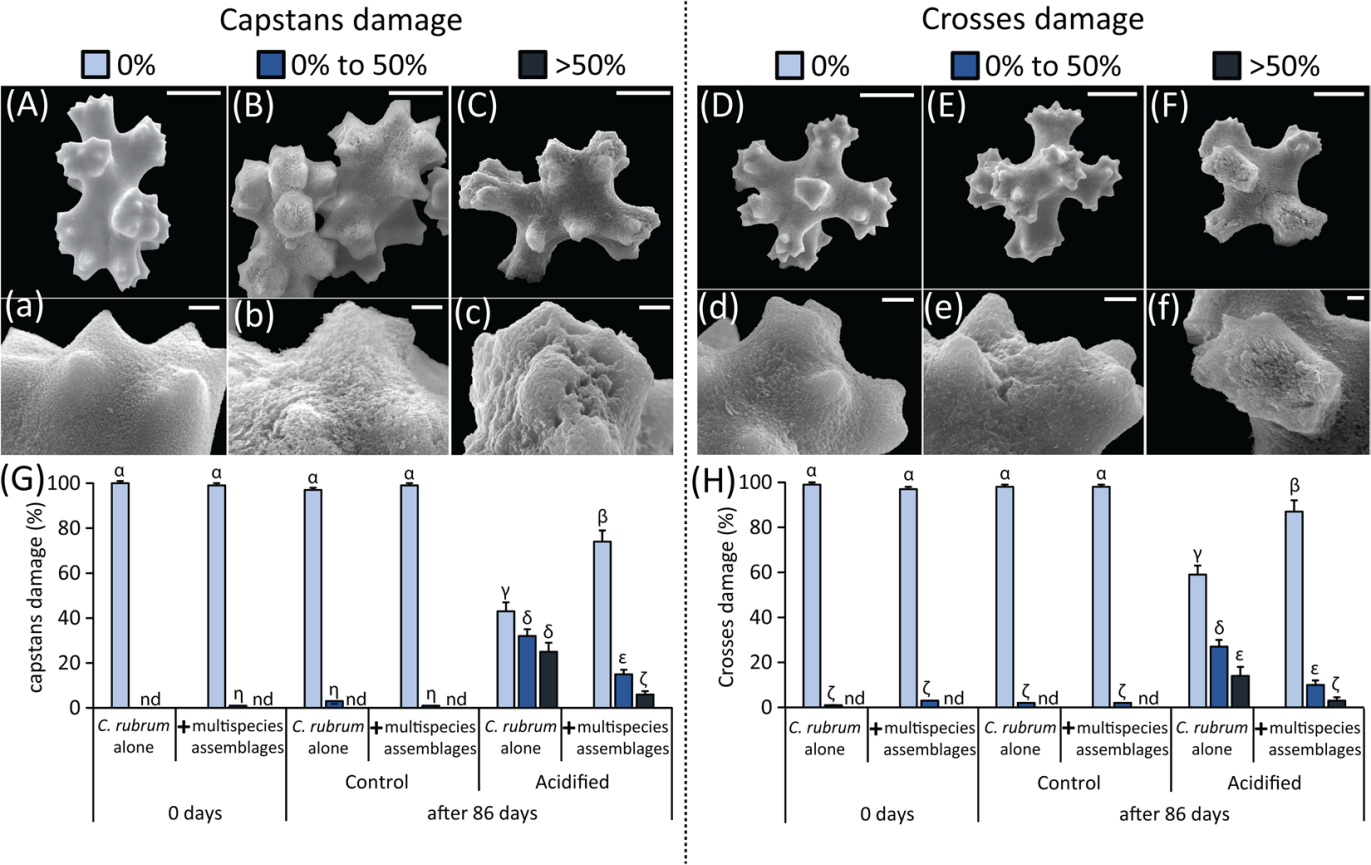
Impact of acidification on the red coral sclerites. Reported are the Scanning Electron Microscopy images of the red coral’s sclerites, including either capstans (**A**–**C** and respective enlarged details a-c) or crosses (**D**–**F** and respective enlarged details d-f), and indicative of different levels of damage ranked (**A**,a and **D**,d: <0% damage; **B**,b and **E**,e: between 0% and 50%; **C**,c and **F**,f: >50%). The bar plots show the frequency of the different levels of damage to red coral capstans (**G**) or crosses (**H**) for the different experimental systems [i.e., acidified and non-acidified control mesocosms, containing *C. rubrum* alone or in association with the coralligenous multispecies assemblages, at the start (0 days) and at the end (86 days) of the experiment]. Reported are average values and SDs (n.d., none detected). Greek letters are used to highlight the significant differences (p < 0.01) among the reported values, with α > β > γ > δ > ε > ζ > η in panel (**G**), and α > β > γ > δ > ε > ζ in panel (**H**).

These results confirm the negative impact of acidification on the red corals when incubated alone^2,82^. Indeed, we found that the acidification treatment caused decalcification of *C. rubrum* calcium carbonate sclerites (57 ± 6% of damaged sclerites compared to no damages in the controls; p < 0.01; Fig. 2) and decrease of its feeding and calcification activities (ranging from −15 to −54% compared to the controls; p < 0.01; Supplementary Fig. S5). In detail, *C. rubrum* polyp’s activity decreased by 44 ± 10% and the percentage of open polyps by 32 ± 11%, while the newly accreted capstans decreased by 34 ± 8% and the crosses by 25 ± 10% (Supplementary Fig. S5). These results are consistent with evidences obtained in previous studies^2^. Indeed, while control levels of polyps’ expansion and sclerites accretion indicate healthy conditions of the colonies, their lower values under acidification suggest a decrease of *C. rubrum* feeding, respiration and calcification efficiency^2,83^. It is well known that calcification in *C. rubrum* is particularly sensitive to seawater acidification^2,3^, likely due to the composition of its skeleton and sclerites (Mg-rich calcite, which is more soluble than other CaCO_3_ forms)^65^ and its apparent inability to up-regulate pH at the site of calcification^82^. Such anomalies in calcification rates and malformation or dissolution of carbonate structures due to seawater acidification are consistent with evidences on tropical corals^11,84,85^.

Moreover, in the acidified systems containing *C. rubrum* alone, the red coral colonies showed a typical physiological adaptation to stressful conditions^2,3,84^, with a preferential production of smaller (crosses) over larger (capstans) coral sclerites (i.e., of +66.8 ± 13.2% compared to the controls; p < 0.01; Supplementary Fig. S5). Indeed, the increased proportion of small crosses has been suggested as an adaptation of the colonies to face the acidified conditions by decreasing the production of larger capstan and skeletal structures and thus limiting the energetic cost of calcification while preserving coenenchimal stiffness^2,3,84^.

Nevertheless, in the present study we show that the negative impacts of acidification were reduced if *C. rubrum* was associated to the natural coralligenous assemblages. In fact, the acidified red coral colonies in this case displayed higher integrity of their calcium carbonate sclerites (i.e., structural damages in only 20 ± 5% of the total sclerites; p < 0.01; Fig. 2). Consistently, *C. rubrum* associated with the natural coralligenous assemblages did not show a decrease of its feeding and calcification activity under acidified conditions (Supplementary Fig. S5). At the same time, *C. rubrum* associated with the natural coralligenous assemblages maintained its ability to keep the cross-to-capstan ratio at the same values of the non-acidified controls (Supplementary Fig. S6).

Previous studies hypothesized that benthic organisms would better contrasts seawater acidification in sediments rich in carbonates, as their dissolution may stabilize pH^86^. As such, we tested if the mitigation of the negative effects of acidification on *C. rubrum* was due to some buffering effect by the carbonatic coralligenous rocks. However, our study does not support this hypothesis, as in the acidified systems the carbonates parameters and pH values showed no significant differences in the mesocosms containing the red coral alone or in association with the coralligenous assemblages (Table 1 and Supplementary Fig. S2). At the same time, the continuous monitoring of the prokaryotic and viral abundances in the mesocosms confirmed the lack of significant differences among the experimental mesocosms, indicating the absence of bacterial or viral pathogenic outbreaks, and excluding that such events could play a role in the differential response to OA (Supplementary Fig. S4).

Therefore, different mechanisms should be invoked to explain the positive effects of the natural coralligenous assemblages on the resistance of *C. rubrum* to acidification.

**Figure 3:**
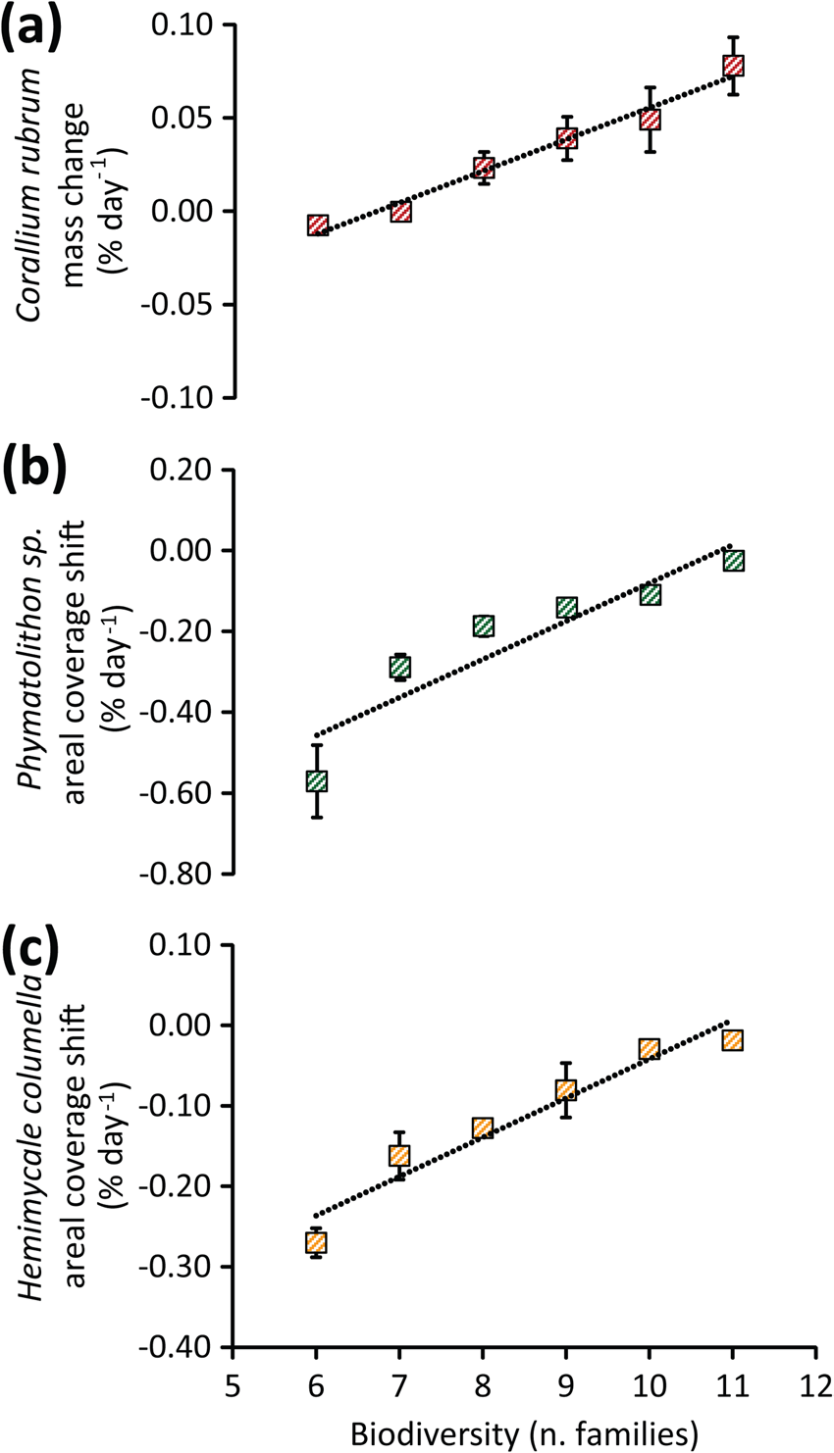
A higher biodiversity reduces the impacts of acidification. For all acidified mesocosms tested in the present study, reported are the positive relationships found between biodiversity (as number of different families contained in the natural coralligenous assemblages tested) and (**a**) the daily changes in mass weight of *C. rubrum* colonies, (**b**) the daily areal coverage shifts of *Phymatolithon* sp. (the dominant coralline alga) and (**c**) the daily areal coverage shifts of *H. columella* (the dominant epilithic sponge). Reported are average values and SDs.

In this regard, our study reports, for the first time, a significant and positive correlation between biodiversity and the species’ resistance to acidification (Fig. 3). In fact, we found that the negative effects of acidification on the red coral (in terms of mass loss due to acidification) decreased with increasing biodiversity of the associated coralligenous assemblages (y = 0.017 × −0.113; R^2^ = 0.976; p < 0.01; Fig. 3a). In detail, under low pH conditions, the red coral growth rates in the most biodiverse assemblages (0.08 ± 0.02% d^−1^) were on average 10 times higher than in the less biodiverse ones (−0.008 ± 0.004% d^−1^) (Fig. 3a).

Increasing biodiversity had positive effects also on the dominant coralline alga *Phymatolithon sp*. and the dominant epilithic sponge *H. columella*. These showed lower impacts of acidification (in terms of decrease in areal coverage) with higher biodiversity of the coralligenous assemblages (y = 0.094 × −1.025 and R^2^ = 0.846; y = 0.0486 × −0.5282 and R^2^ = 0.936 for the alga and the sponge, respectively; both p < 0.01; Fig. 3b,c). In detail, under acidified conditions, the decrease in areal coverage of *Phymatolithon sp*. in the most biodiverse assemblages (−0.03 ± 0.01% d^−1^) was on average 22 times lower than in the less biodiverse ones (−0.57 ± 0.09% d^−1^). While for *H. columella*, the decrease in areal coverage in the most biodiverse assemblages (−0.02 ± 0.01% d^−1^) was on average 14 times lower than in the less biodiverse ones (−0.27 ± 0.02% d^−1^).

Notably, a higher biodiversity was associated with higher growth rates of the dominant coralligenous taxa also in the non-acidified controls (Supplementary Fig. S7), indicating that this feature was consistent across control and acidified treatments. In detail, under control conditions, the growth rates of *C. rubrum*, *Phymatolithon sp*. and *H. columella* in the most biodiverse assemblages were respectively 1.4, 7.2 and 9.2 times higher on average than in the less biodiverse ones (0.14 ± 0.01 *vs* −0.010 ± 0.001% d^−1^ for *C. rubrum*; 0.15 ± 0.04 *vs* −0.02 ± 0.01% d^−1^ for *Phymatolithon sp.;* 0.09 ± 0.03 *vs* −0.010 ± 0.005% d^−1^ for *H. columella*). Overall, these results indicate that, at the highest biodiversity tested in our study, the impact of acidification on otherwise highly vulnerable key organisms could be reduced by ca. 50 to >90%, depending on the species (Fig. 3a–c).

Several factors can contribute to explain such observed positive effects of increasing biodiversity in the fostering of the species’ resistance to acidification. On one hand, our results show that higher biodiversity was associated with higher availability of organic matter (R^2^ = 0.844; p < 0.01; Supplementary Fig. S8). Such positive link between biodiversity and trophic enrichment is consistent with previous evidences, which show the presence of highly biodiverse coralligenous structures to increase the benthic availability of trophic resources^87,88^, as well as with reports of higher biodiversity in hard-bottom ecosystems associated to higher organic matter contents^89^. As organic matter is a fundamental source of energy in the diet of *C. rubrum* (also supporting calcification)^90,91^ and sponges^92,93^, its higher availability in more biodiverse multispecies assemblages might contribute to explain the enhanced feeding activity and efficiency of the red coral and sponges and hence their higher resistance to acidification as observed in the present study.

**Figure 4:**
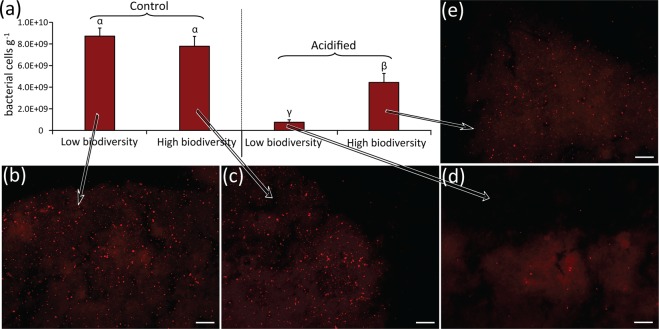
Impact of acidification on the association between the sponge *Hemimycale columella* and calcibacteria. The bar plot (**a**) reports the number of bacterial cells per gram of sponge dry weight, as determined by CARD-FISH on calcibacteria extracted from fresh *H. columella* sponge tissues. Average values and standard deviations are shown for samples collected at the end of the experiment (86 d) from acidified and non-acidified mesocosms containing low-biodiversity assemblages (i.e., only 6 families), or high-biodiversity assemblages (i.e., 11 families). The epifluorescence microscopy images show sections of the corresponding samples of *H. columella* sponge tissues from which calcibacteria were extracted, analyzed in parallel by CARD-FISH. (**b**), control (low-biodiversity, 6 families); (**c**), control (high-biodiversity, 11 families); (**d**), Acidified (low-biodiversity, 6 families); E, acidified (high-biodiversity, 11 families). Scale bar: 40 µm. Greek letters are used to highlight the significant differences (p < 0.01) among the reported values, with α > β > γ.

On the other hand, our study indicates that a higher biodiversity can favour and stabilise the cooperation between the large species of the coralligenous assemblages and their associated microbes and that this, in turn, can have positive effects on the species’ resistance to acidification. In fact, we report here that exposing the dominant demosponge species in our coralligenous assemblages (*H. columella*) to acidification caused a massive loss of the calcifying bacterial symbionts associated to its tissues (p < 0.01; Fig. 4). Nevertheless, in the more biodiverse assemblages, the loss of calcifying symbionts was much lower (p < 0.01; Fig. 4), suggesting a possible role of biodiversity in fostering a higher stability of host-microbe interactions that can increase host resistance to acidification. Our results thus add new evidence that sponges can thrive upon complex associations and symbioses with microbes^93,94^, and that preserving healthy microbiomes in highly biodiverse multispecies assemblages can contribute to increase the sponge resistance to acidification^40,95,96^.

**Figure 5:**
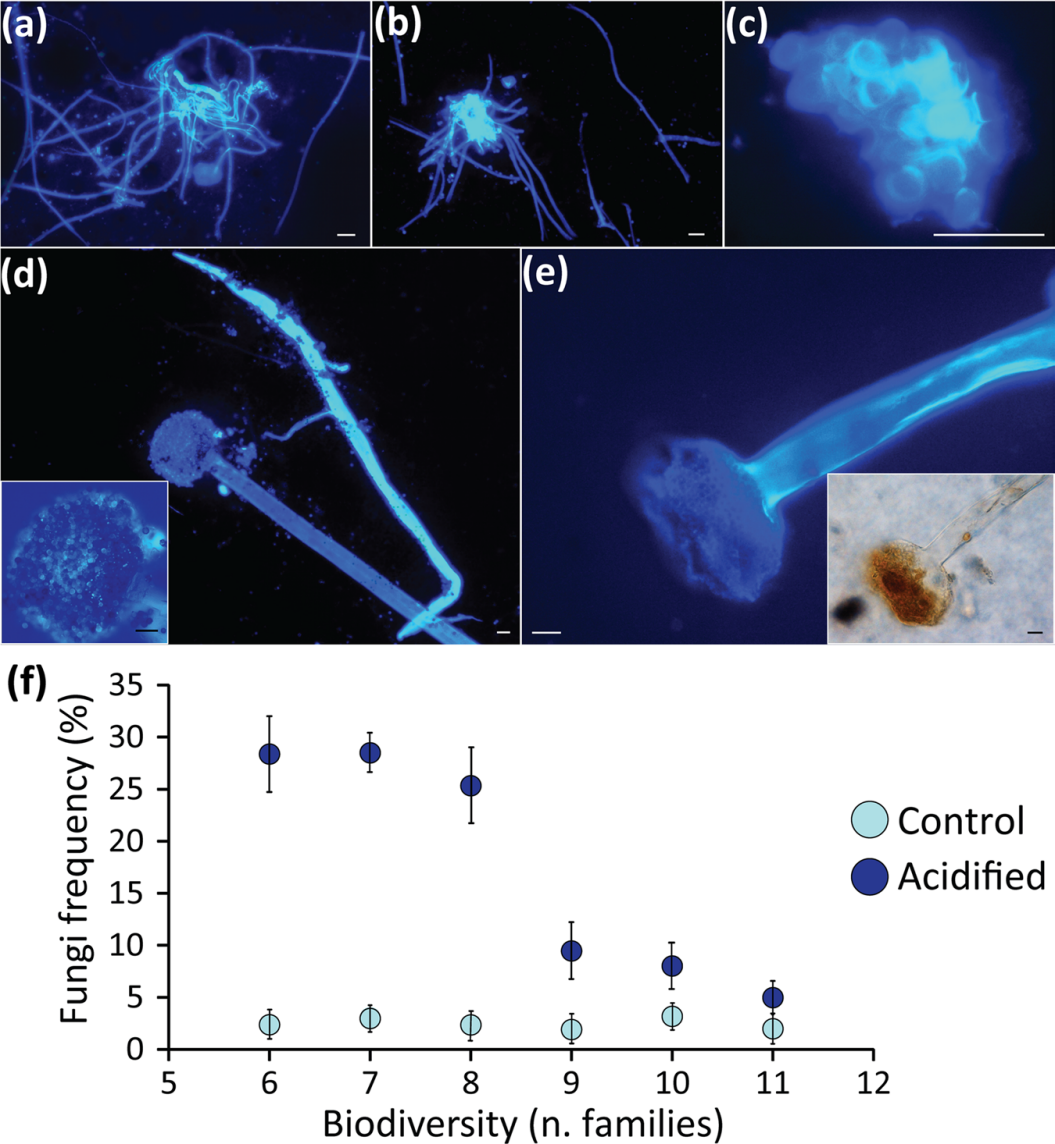
Impact of acidification on unicellular fungi. Reported are microscopy images, illustrating examples of fungal structures colonizing the surface of the coralligenous assemblages tested in the present study. (**a**,**b**) fungal hyphae with septa and filamentous branching; (**c**) cluster of fungal spores; (**d**,**e**) sporangia with enlarged highlights showing details of the spores under epifluorescence or normal light microscopy (scale bars, 10 µm). The scatter plot (**f**) shows the relationships between biodiversity (as number of different families contained in the natural coralligenous assemblages tested) and the proliferation of fungi in acidified and non-acidified (control) mesocosms. Reported are average values and SDs.

Recent evidences suggest that also unicellular fungi can be involved in complex networks of microbe-host interactions that can influence the responses of the sessile habitat-forming species to acidification^97^. In the present study, we only occasionally detected fungi on the surface of the coralligenous substrate at the start of the experiments (Fig. 5). However, under acidified conditions, we reported a clear fungal proliferation, with frequency of fungal detection on average 7 and up to >30 higher compared with the controls (p < 0.01; Fig. 5).

This is consistent with current evidence showing that marine fungi can be favoured by acidification^98,99^. Notably, the proliferation of fungi decreased with increasing biodiversity (R^2^ = 0.847; p < 0.01; Fig. 5), suggesting that the more biodiverse coralligenous assemblages better contrasted the observed outbreak of opportunistic fungi (possibly including parasitic/pathogenic strains^100,101^. In Octocorallia, including the red coral *C. rubrum*, the sclerites can play a key role as physical and chemical barriers against fungal infection^102^. Therefore, our results suggest that biodiversity loss, coupled with reduced production of coral sclerites, can make the red coral more vulnerable to fungal infection, thus exacerbating the negative effects of OA on this species. Possible links between the proliferation of fungi under acidified conditions and the loss of calcifying bacteria reported in our study for *H. columella*, as well as with the observed decreased in the areal coverage of the dominant macroalgae might be hypothesized^97^ and deserve further investigation.

Another factor that can contribute to the increase of resistance to acidification at high biodiversity levels, is the fact that a higher biodiversity is associated to a higher functional redundancy^26–29^. Even if not specifically tested in the present study, we hypothesize that, the higher the biodiversity, the higher the probability of functional redundancy among species (considering either macro-, meio-and/or micro-organisms) which in turn can reduce the possibility to lose specific assemblage functions.

Finally, recent evidence suggested that, in coral reefs, the biogenic dissolution of carbonates by microbial borers (including endolithic bacteria, microalgae and fungi) may be a significant driver of carbonate dissolution in low pH conditions^103^, and this can be observed either in carbonatic substrates and on the skeleton/structures of living organisms^104^. We thus hypothesize that OA can not only alter microbe-host interactions but can also influence the activity of microborers, thus exacerbating the impact of acidification on the calcifying species.

Overall, our results agree with ecological theories predicting that a higher biodiversity promotes higher stability and resistance to environmental changes^16,26–29,105^, as well as with experimental evidences that, in biodiversity-rich assemblages, microbe-host interactions can be more stable than in low-biodiversity assemblages, due to the increased potential functional redundancy^40,42,106^. Our study supports the perspective that the complex networks of biotic interactions occurring between microbes and large sessile species in multispecies assemblages can significantly influence the species’ response to environmental alterations brought on by global change^17,28–31,40,42,106,107^. We show here that a higher biodiversity and healthy microbe-host interactions, can largely contribute to mitigate the negative impacts of acidification on otherwise highly vulnerable species. These results support the current perspective that marine conservation actions help the oceans to mitigate and adapt to climate change by promoting intact and complex ecosystems with high diversity and abundance of species^108–113^. Despite the understanding of the specific mechanisms underlying the observed positive effects of a high biodiversity deserve further investigation, our timely results allow highlighting that the biodiversity conservation of hard-bottom ecosystems will be crucial in the future also to increase their stability and resistance to the threats of OA.

## Supplementary information

Supplementary Figures

## Data Availability

All data of this study are included in the present manuscript and its Supplementary Information file.
