# Supplementary material for: A high biodiversity mitigates the impact of ocean acidification on hard-bottom ecosystems
Source: Sci Rep. 2020 Feb 19;10:2948. doi: 10.1038/s41598-020-59886-4 (PMC7031329; doi:10.1038/s41598-020-59886-4)
Supplement: Supplementary file 1 — Supplementary Figures [file 41598_2020_59886_MOESM1_ESM.docx]

**Supplementary Information**

**A high biodiversity mitigates the impact of ocean acidification on hard-bottom ecosystems**

Eugenio Rastelli, Bruna Petani, Cinzia Corinaldesi, Antonio Dell’Anno, Marco Lo Martire, Carlo Cerrano, Roberto Danovaro

**This file includes:**

**Supplementary Figures S1-S8**


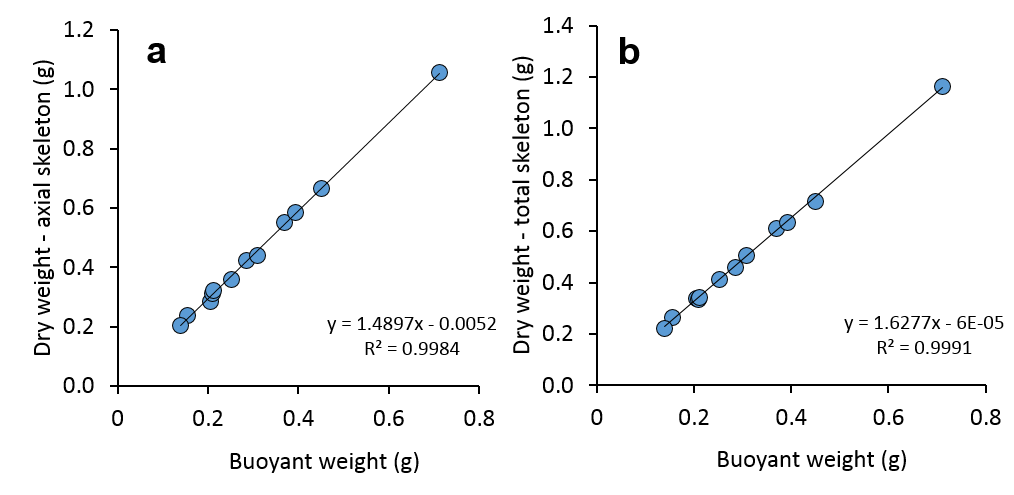


(A)

(B)

**Supplementary Figure S1. Relationship between coral colonies’ buoyant and dry weight.** Illustrated are relationship between colonies’ buoyant weight and axial skeleton dry weight (A) and relationship between colonies’ buoyant weight and total skeleton dry weight (i.e., scleraxis plus sclerites) (B).


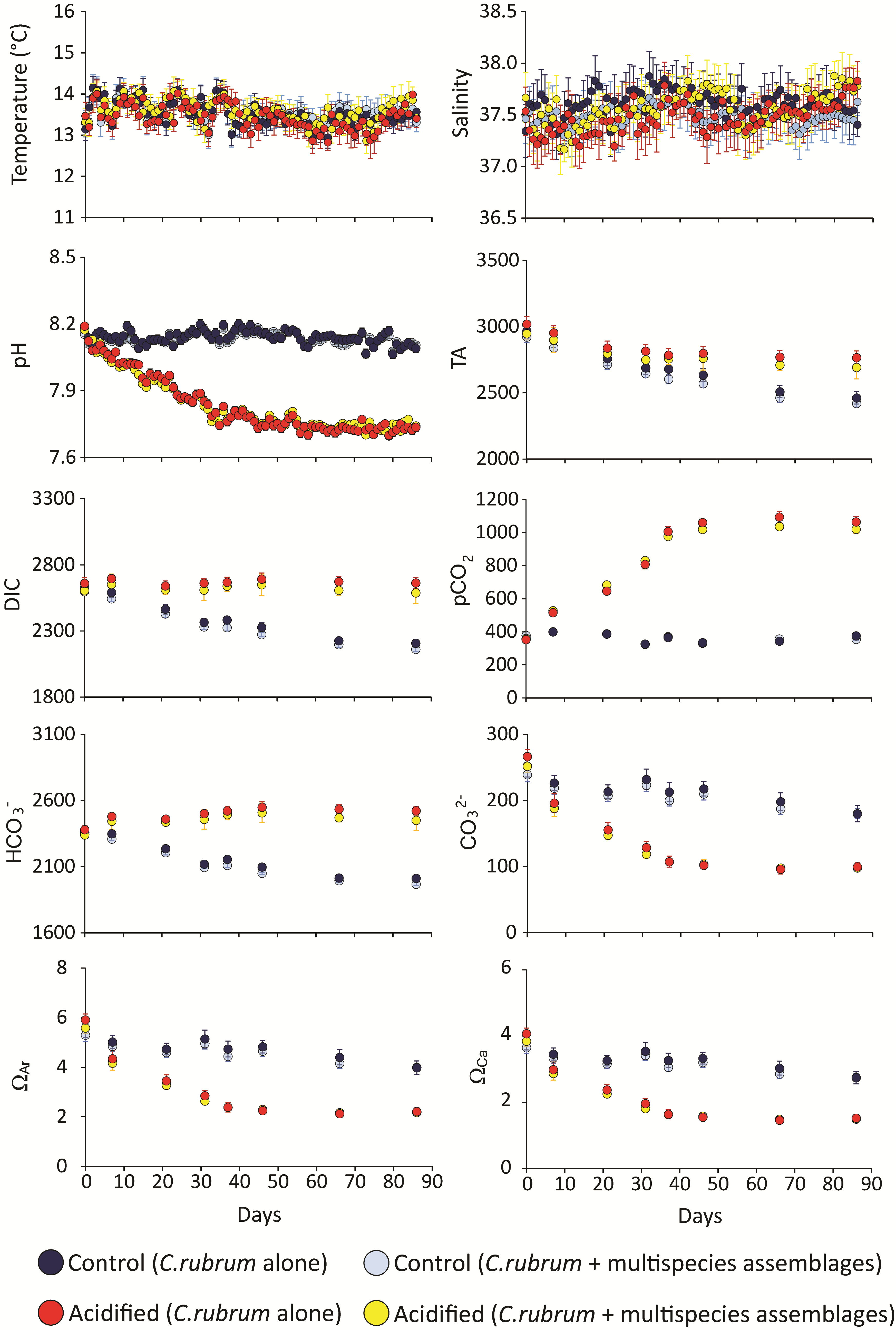


**Supplementary Figure S2. Physical-chemical variables and carbonate parameters measured in control and acidified treatments.** For the controls (treated at 400 ppm CO_2_) and acidified treatments (i.e., acidified at 1000 ppm of CO_2_ to reach the desired pH of approx. 7.7), reported are mean values ± standard deviations. S, salinity; T, temperature; TA, total alkalinity (µmol kg^-1^); DIC, dissolved inorganic carbon (µmol kg^-1^); pCO_2_ partial pressure of carbon dioxide (µatm); HCO_3_^-^ , bicarbonates (µmol kg^-1^); CO_3_^2-^, carbonates (µmol kg^-1^); Ω_Ar_, aragonite saturation state; Ω_Ca_ calcite saturation state.


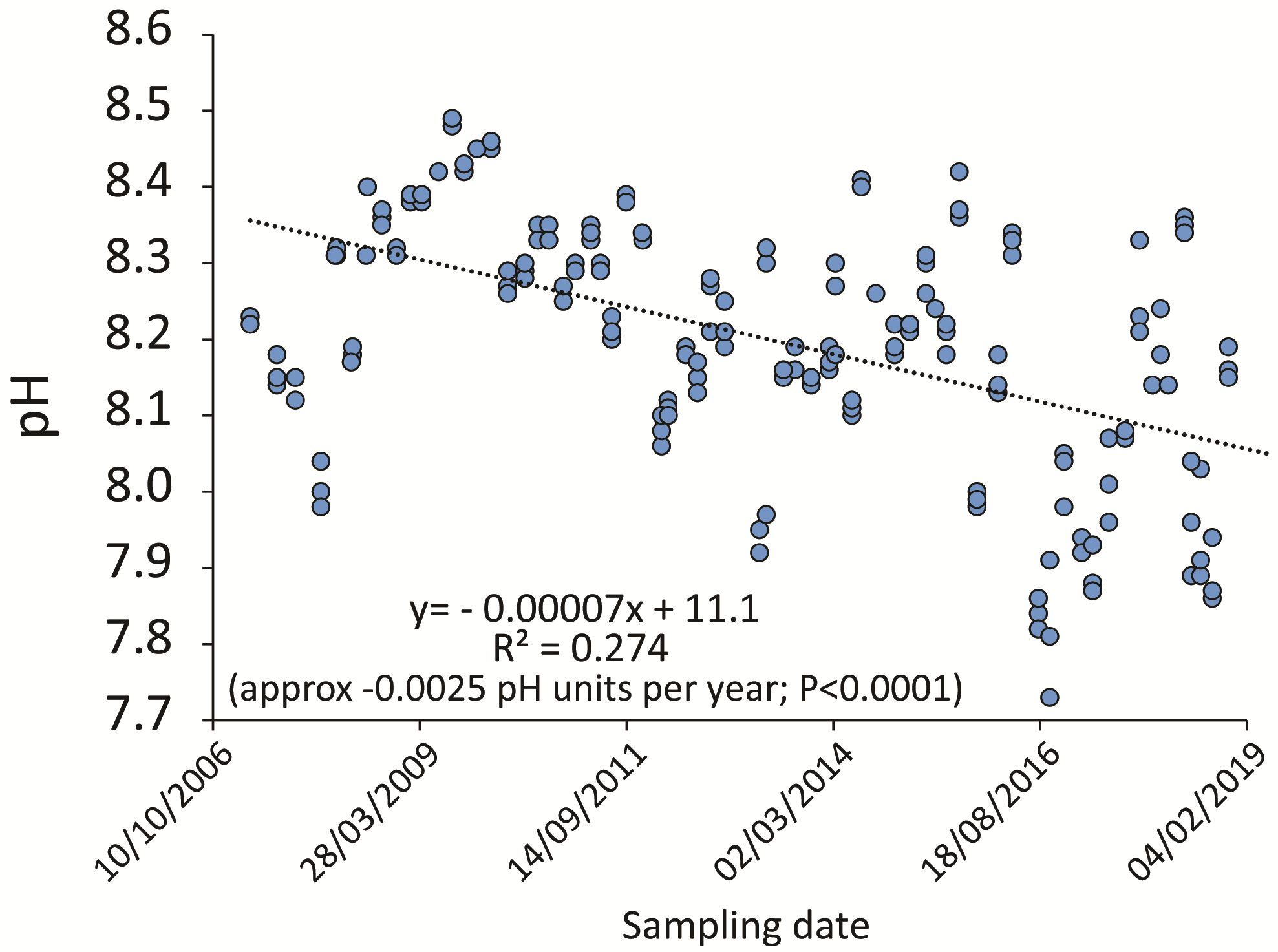


**Supplementary Figure S3. Historical dataset for the values of pH in the study area.** Reported are values of pH for the water mass at 30 m below the sea surface for the area in which the organisms used in this study were sampled. Data are from the long-term official database available online at the Ligurian Region (http://rgetrasweb.regione.liguria.it/qpg/Tree.do?codNodo=3282).

(A)


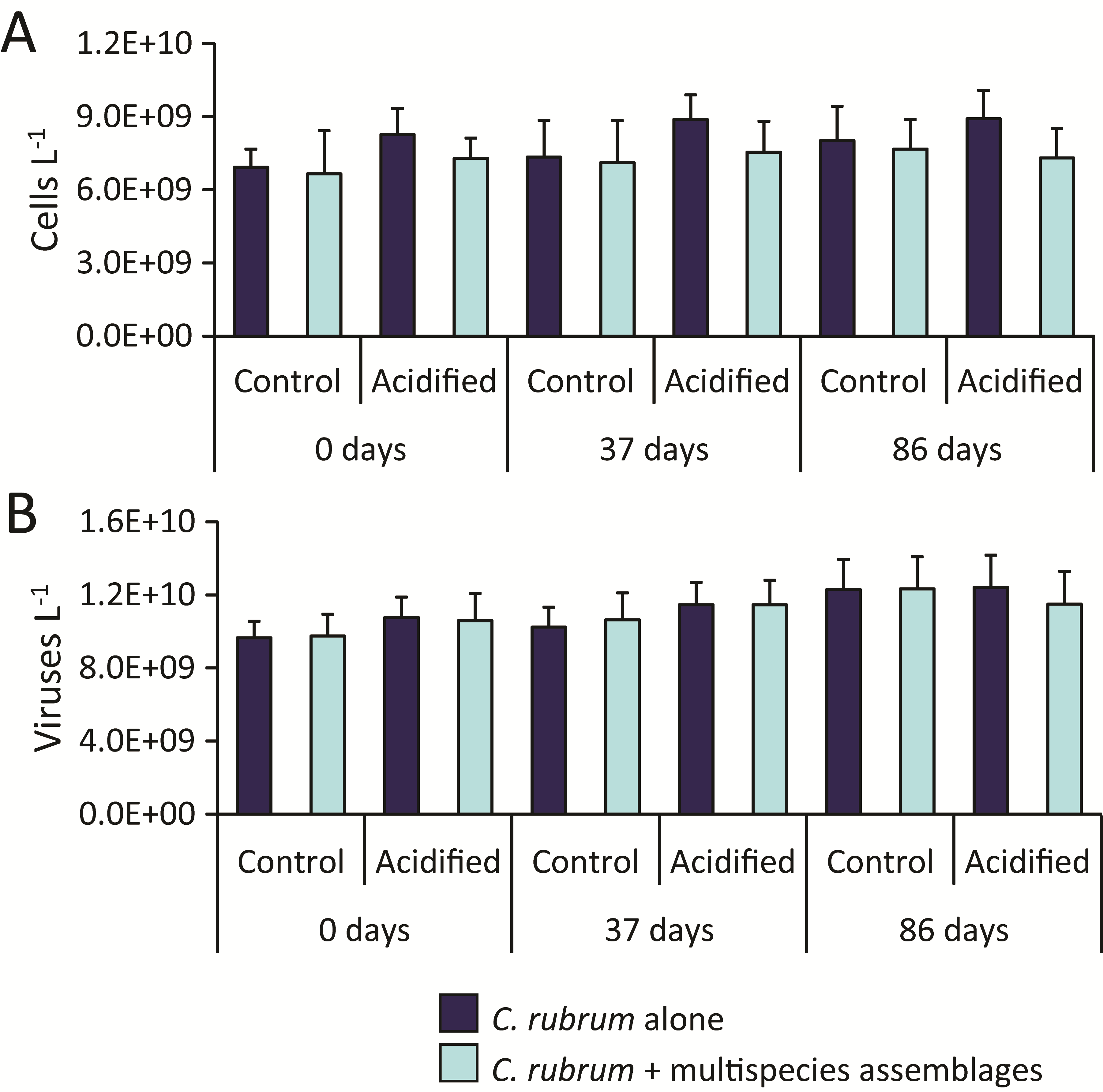


(B)

**Supplementary Figure S4. Prokaryotes and virus abundances in the control and acidified treatments during the experiment.** Reported are the prokaryotic (A) and viral (B) abundances for the different experimental systems (i.e., control and acidified treatments, containing *C. rubrum* alone or in association with the multispecies coralligenous assemblages) at the start of the experiment (i.e., 0 days), after 37 days, and at the end of the experiment (i.e., 86 days). Reported are average values and SDs.


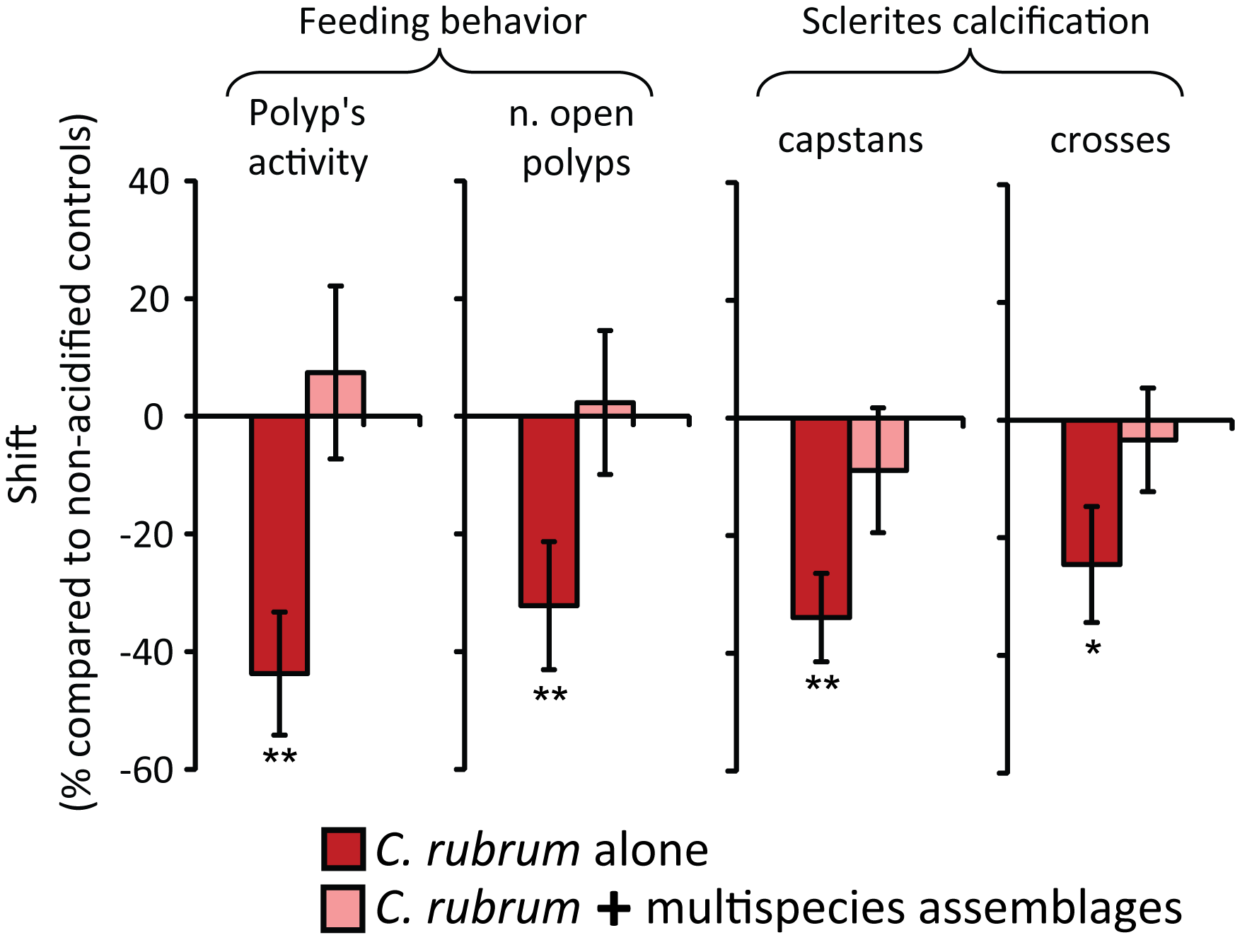


**Supplementary Figure S5. Impact of acidification on the feeding behavior and calcification processes of *C. rubrum* for systems containing the red coral alone or in association with multispecies assemblages.** Illustrated are the functional changes in the red coral due to acidification, in terms of feeding behavior (estimated based on the polyps’ activity and the number of open polyps) and of calcification processes (based on the quantification of the newly-accreted capstans and crosses). Reported are average values and SDs. Asterisks indicate significant differences found when comparing the values reported for the two types of acidified systems (i.e., the mesocosms containing C rubrum alone or in association with the coralligenous assemblages); with “*” for p<0.05 and “**” for p<0.01. The acidified systems containing the red coral in association with multispecies assemblages did not show significant shifts compared to the non-acidified controls, as indicated by shift values overlapping zero and p>0.05.


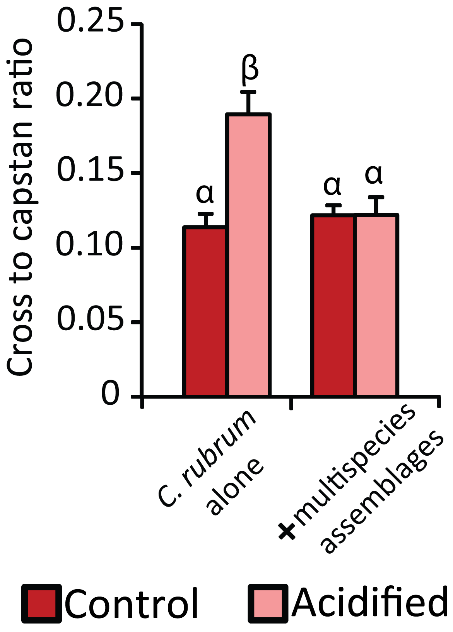


**Supplementary Figure S6.** **Impact of acidification on calcification processes of *C. rubrum* for systems containing *C. rubrum* alone or in association with multispecies assemblages.** Illustrated are the ratios between the two types of newly-accreted sclerites of the red coral in the different mesocosms analyzed. Reported are average values and SDs. Crosses are typically smaller than the larger capstan sclerites (see also Figure 2 in the main text for SEM images of the two different types of sclerites). Greek letters are used to highlight the significant differences (p<0.01) among the reported values, with β>α.


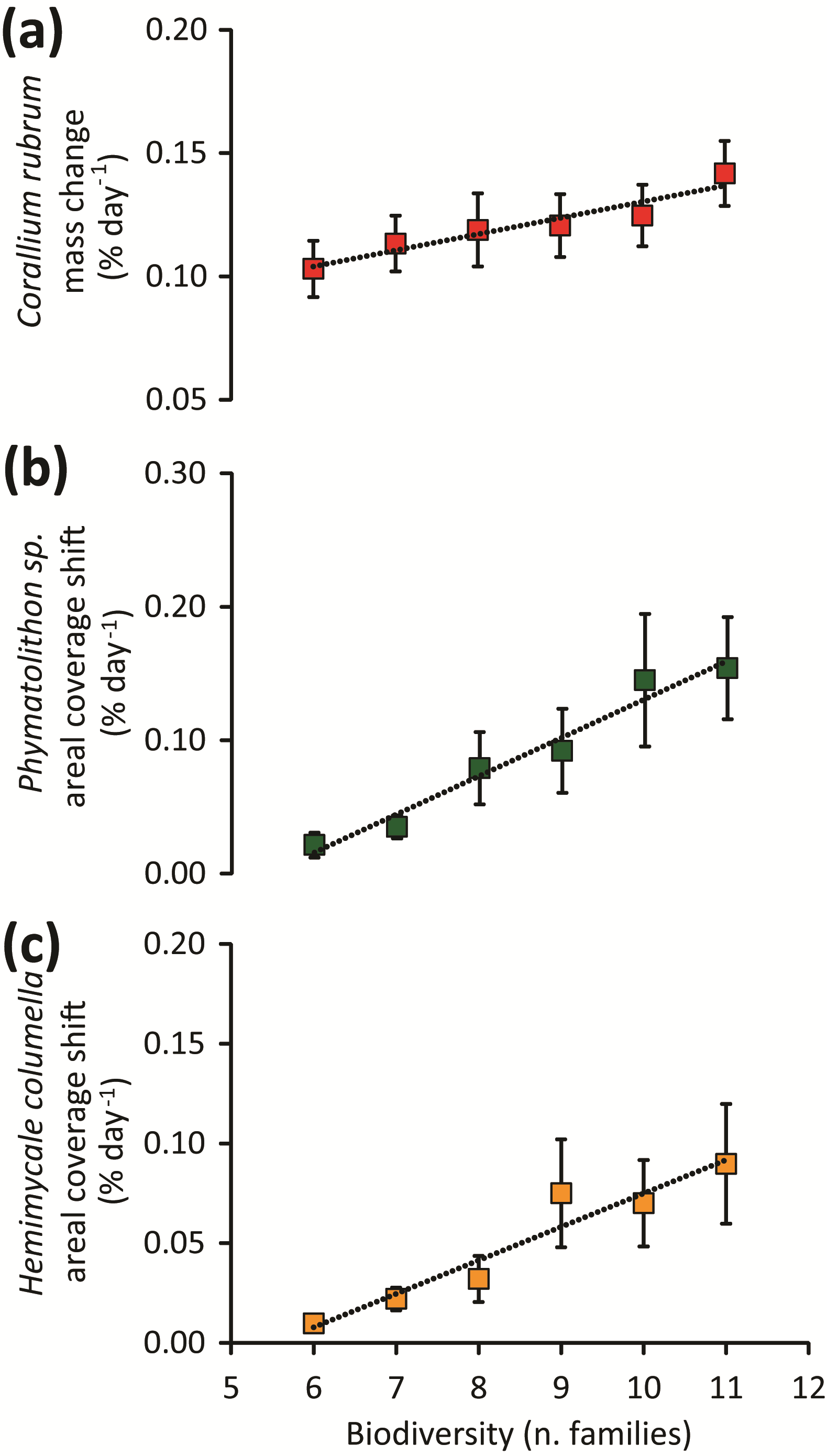


**Supplementary Figure S7.** **Positive effects of increasing biodiversity on the non-acidified coralligenous taxa** . For all control (non-acidified) mesocosms tested in the present study, reported are the positive relationships found between biodiversity (as number of different families contained in the natural coralligenous assemblages tested) and (A) the daily changes in mass weight of *C. rubrum* colonies, (B) the daily areal coverage shifts of *Phymatolithon sp.* (the dominant coralline alga) and (C) the daily areal coverage shifts of *H. columella* (the dominant epilithic sponge). Reported are average values and SDs.


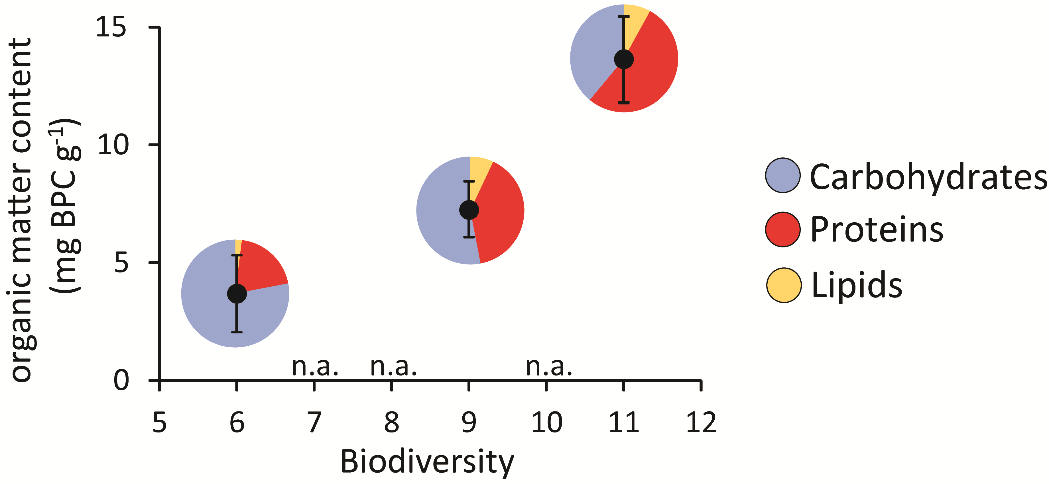


**Supplementary Figure S8.** **Relationships between biodiversity and organic matter contents.** Reported is the positive relationship found between the level of biodiversity (as number of different families contained in the natural coralligenous assemblages) and the availability of organic matter (as biopolymeric organic C, BPC, composed of carbohydrates, proteins and lipids) in the analyzed systems. n.a., data not available.
